# The role of the acyl-CoA thioesterase “YciA” in the production of (*R*)-3-hydroxybutyrate by recombinant *Escherichia coli*

**DOI:** 10.1007/s00253-019-09707-0

**Published:** 2019-03-05

**Authors:** Mónica Guevara-Martínez, Mariel Perez-Zabaleta, Martin Gustavsson, Jorge Quillaguamán, Gen Larsson, Antonius J. A. van Maris

**Affiliations:** 1 Department of Industrial Biotechnology, School of Engineering Sciences in Chemistry, Biotechnology and Health, KTH Royal Institute of Technology, AlbaNova University Center, SE 10691 Stockholm, Sweden; 2 Faculty of Science and Technology, Center of Biotechnology, Universidad Mayor de San Simón, Cochabamba, Bolivia

**Keywords:** *Escherichia coli*, *Halomonas boliviensis*, (*R*)-3-hydroxybutyrate, Thioesterase, *yciA*

## Abstract

Biotechnologically produced (*R*)-3-hydroxybutyrate is an interesting pre-cursor for antibiotics, vitamins, and other molecules benefitting from enantioselective production. An often-employed pathway for (*R*)-3-hydroxybutyrate production in recombinant *E. coli* consists of three-steps: (1) condensation of two acetyl-CoA molecules to acetoacetyl-CoA, (2) reduction of acetoacetyl-CoA to (*R*)-3-hydroxybutyrate-CoA, and (3) hydrolysis of (*R*)-3-hydroxybutyrate-CoA to (*R*)-3-hydroxybutyrate by thioesterase. Whereas for the first two steps, many proven heterologous candidate genes exist, the role of either endogenous or heterologous thioesterases is less defined. This study investigates the contribution of four native thioesterases (TesA, TesB, YciA, and FadM) to (*R*)-3-hydroxybutyrate production by engineered *E. coli* AF1000 containing a thiolase and reductase from *Halomonas boliviensis*. Deletion of *yciA* decreased the (*R*)-3-hydroxybutyrate yield by 43%, whereas deletion of *tesB* and *fadM* resulted in only minor decreases. Overexpression of *yciA* resulted in doubling of (*R*)-3-hydroxybutyrate titer, productivity, and yield in batch cultures. Together with overexpression of glucose-6-phosphate dehydrogenase, this resulted in a 2.7-fold increase in the final (*R*)-3-hydroxybutyrate concentration in batch cultivations and in a final (*R*)-3-hydroxybutyrate titer of 14.3 g L^−1^ in fed-batch cultures. The positive impact of *yciA* overexpression in this study, which is opposite to previous results where thioesterase was preceded by enzymes originating from different hosts or where (*S*)-3-hydroxybutyryl-CoA was the substrate, shows the importance of evaluating thioesterases within a specific pathway and in strains and cultivation conditions able to achieve significant product titers. While directly relevant for (*R*)-3-hydroxybutyrate production, these findings also contribute to pathway improvement or decreased by-product formation for other acyl-CoA-derived products.

**Electronic supplementary material:**

The online version of this article (10.1007/s00253-019-09707-0) contains supplementary material, which is available to authorized users.

## Introduction

Production of valuable renewable chemicals and fuels via bio-based processes provides an alternative to petroleum-based processes (Lee et al. 2011, 2012). For an efficient switch to bio-based refineries, it is essential to employ metabolic engineering to improve pathways and increase product diversity. In addition to production from sustainable resources, bio-based production of enantiomerically pure chiral molecules has the benefit of the enantioselectivity of enzymes and operation at ambient temperatures and atmospheric pressures (Patel 2006; Pollard and Woodley 2007). Enantiomers of hydroxy carboxylic acids have potential applications as building blocks for the synthesis of many compounds such as antibiotics and various copolymers (Ren et al. 2010). One such example is the chiral molecule (*R*)-3-hydroxybutyrate (3HB). This molecule has important applications as precursor for the synthesis of antibiotics and vitamins (Chiba and Nakai 1985; Chiba and Nakai 1987; Seebach et al. 1986). Its dimers and trimers have been considered as precursor of ketone bodies for nutritional care in eukaryotic cells (Tasaki et al. 1999). Furthermore, 3HB can be used as building block for synthesis of various polyhydroxyalkanoates (PHA), a family of polyesters with a wide variety of qualities and applications (Anderson and Dawes 1990).

**Fig. 1:**
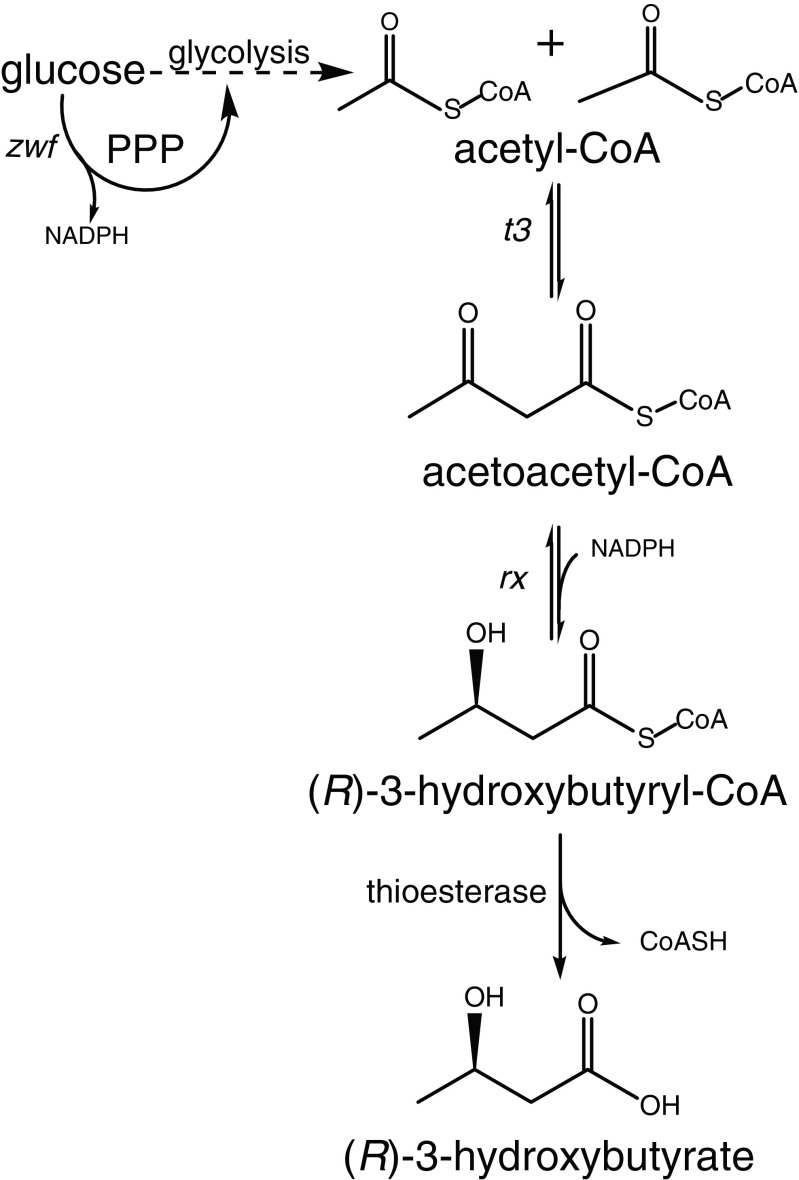
Schematic overview of 3HB pathway for production in recombinant *E. coli.* 3HB production from glucose starts with glycolysis to produce acetyl-CoA. Subsequently, there is a three-step conversion: (1) condensation of two molecules of acetyl-CoA to acetoacetyl-CoA catalyzed 3-keto-thiolase, (2) reduction of acetoacetyl-CoA to (*R*)-3HB-CoA catalyzed by acetoacetyl-CoA reductase, and (3) hydrolysis of (*R*)-3HB-CoA to 3HB catalyzed by a thioesterase and subsequent export to the medium. In this study, genes coding enzymes acetoacetyl-CoA thiolase (*t3*) and acetoacetyl-CoA reductase *(rx)* were cloned from *H. boliviensis* and expressed in *E. coli* AF1000

3HB can be produced in different ways: via chemical catalysis (Jaipuri et al. 2004; Noyori et al. 1987), via enzymatic or chemical degradation of polyhydroxybutyrate (PHB) (de Roo et al. 2002; Lee et al. 1999, 2000), or via fermentation with metabolically engineered microorganisms (Gao et al. 2002; Gulevich et al. 2017; Lee and Lee 2003; Liu et al. 2007; Matsumoto et al. 2013; Tseng et al. 2009). Direct production of 3HB from renewable raw materials using engineered strains is a promising approach that also avoids the extreme conditions required for chemical catalysis or the use of two consecutive processes for the route through PHB. Microorganisms can produce 3HB from the central metabolite acetyl-CoA through a three-step conversion (Fig. 1): (1) condensation of two molecules of acetyl-CoA to acetoacetyl-CoA catalyzed by 3-keto-thiolase, (2) stereospecific reduction of acetoacetyl-CoA to (*R*)-3HB-CoA catalyzed by acetoacetyl-CoA reductase, and (3) hydrolysis of (*R*)-3HB-CoA to 3HB by thioesterase and subsequent export to the medium.

Microbial production of 3HB has previously been achieved by heterologous expression of pathway genes in *E. coli* (Gao et al. 2002; Guevara-Martínez et al. 2015; Jarmander et al. 2015; Lee and Lee 2003; Liu et al. 2007; Perez-Zabaleta et al. 2016; Tseng et al. 2009). *Halomonas boliviensis* is a halophilic bacteria known to accumulate PHB (Quillaguaman et al. 2008; Quillaguaman et al. 2004). In previous work, we heterologously expressed thiolase 3 (*t3*) and reductase x (*rx*) from *H. boliviensis* in *E. coli* strain AF1000, thereby enabling the first two steps for conversion of acetyl-CoA to 3HB-CoA (Fig. 1) (Guevara-Martínez et al. 2015; Jarmander et al. 2015; Perez-Zabaleta et al. 2016). Importantly, no heterologous gene encoding a thioesterase was introduced in those studies, assuming sufficient activity of unspecified native thioesterases. In combination with further engineering of NADPH provision, through overexpression of the native glucose-6-phosphate dehydrogenase encoded by “*zwf*”*,* the engineered *t3*-*rx*-based *E. coli* strain produced up to 12.7 g L^−1^ in 30 h of cultivation (Perez-Zabaleta et al. 2016). Interestingly, in a study on the heterologous expression of *Cupriavidus necator* genes encoding a thiolase and reductase in *E. coli* strain (Fig. 1), Liu et al. (2007) showed that overexpression of the native thioesterase *tesB* was essential in strain DH5α and resulted in a titer of 12.2 g L^−1^ after 24 h of cultivation in strain BW25113.

Improved understanding of the role and identity of the native thioesterase(s) responsible for hydrolysis of 3HB-CoA to 3HB is not only directly relevant for further strain improvement, but also to potentially avoid 3HB as a by-product when other carboxylic acids derived from CoA intermediates are the desired product. Thioesterases (EC 3.1.2.-) are a large group of enzymes which hydrolyze the thioester bond between a carbonyl group and a sulfur atom from a wide class of compounds, such as coenzyme A (CoA), acyl carrier proteins (ACP), glutathione, or other protein molecules (Cantu et al. 2010). Acyl-CoA thioesterases (not to be confused with ACP-thioesterases) are enzymes that catalyze the hydrolysis of acyl-CoAs to the free carboxylic acid and CoA by cleaving the thioester bond of acyl-CoA intermediates (Hunt and Alexson 2002; Tillander et al. 2017), and were first detected in *E. coli* by Kass et al. (1967). The highly negative free-energy change of this reaction also provides a thermodynamic pull on the engineered 3HB production pathway. The genome of *E. coli* encodes multiple candidate thioesterases with diverse roles in metabolism, which have been extensively reviewed (Cantu et al. 2010). Firstly, thioesterase TesA is located in the periplasmic space of *E.coli* and has been reported to increase carboxylic acid production when overexpressed in the cytosol (Klinke et al. 1999). Secondly, thioesterase TesB, a native *E. coli* enzyme, has been reported to hydrolyze β-hydroxyacyl-CoA thioesters (Barnes and Wakil 1968; Barnes et al. 1970). Enhanced productivity of both enantiomers (R and S) of 3HB was reported by overexpression of *tesB* in recombinant *E. coli* (Gao et al. 2002; Gulevich et al. 2017; Liu et al. 2007; Tseng et al. 2009). This enzyme was also reported to play an important role in 3-hydroxydecanoyl-CoA hydrolysis (Zheng et al. 2004). Thirdly, thioesterase FadM is a long chain acyl-CoA thioesterase that plays a role in the β-oxidation of oleic acid, by hydrolyzing the minor side product 3,5-tetradecadienoyl-CoA (Ren et al. 2004). Lastly, a kinetic characterization of the native thioesterase, YciA, revealed that this enzyme exhibits significant catalytic efficiency for many potential acyl-CoA intermediates, including acetyl-CoA, acetoacetyl-CoA, and both (*R*)- and (*S*)-3HB-CoA (Clomburg et al. 2012).

The aim of this study is to identify the native thioesterase(s) responsible for hydrolysis of 3HB-CoA to 3HB in an engineered *E. coli* AF1000 strain expressing the thiolase *t3* and reductase *rx* from *H. boliviensis*. In view of their broad substrate specificity, the *E. coli* thioesterases encoded by *tesA, tesB, yciA*, and *fadM* were selected as candidate thioesterases. Initially, their contribution to 3HB-CoA hydrolysis was investigated by deleting each thioesterase individually in a 3HB-producing strain background. Subsequently, the overexpression of the identified most contributing thioesterase on 3HB production was investigated. To improve 3HB production, the thioesterase was overexpressed in conjunction with glucose-6-phosphate dehydrogenase Zwf and quantitatively assessed in fed-batch cultivations.

## Materials and methods

### Strains and plasmids

The *E. coli* strain background used in this work for 3HB production was AF1000 (Sandén et al. 2003), a *relA+* mutant of MC4100 (ATCC35695). Single gene knockouts were done in strain *E. coli* AF1000, following the protocol described by Jensen et al.(2015) with the modification that cells were directly plated overnight on Luria Bertani (LB) agar plates, which were prepared according to Miller (1972) and contained 50 mM L-rhamnose (Sigma-Aldrich, St Louis, MO) for removal of the antibiotic marker. All deletions were confirmed by PCR and further DNA sequencing. The template plasmid used for the FRT-flanked cat cassette was plasmid pCmFRT*, which is a modified version of pKD3 (Datsenko and Wanner 2000) with stop codons instead of either start codons or RBSs in the six reading frames in between the FRT sites. The plasmid used for either lambda Red recombinase genes or flippase recombinase expression was the temperature-sensitive pSIJ8 (Jensen et al. 2015). All the resulting strains used in this study are listed in Table 1. Primers synthesized by Integrated DNA Technologies (IDT, Leuven, Belgium) used for gene deletion are all listed in Table S1 in Supplementary Material.

**Table 1:** Strains and plasmids used in this study

Strain/plasmid	Description/genotype	Source
AF1000	MC4100, *relA+*	Sandén et al. (2003)
AF1000 ∆*tesA*	AF1000, ∆*tesA*::FRT	This study
AF1000 ∆*tesB*	AF1000, ∆*tesB*::FRT	This study
AF1000 *∆fadM*	AF1000, *∆fadM*::FRT	This study
AF1000 *∆yciA*	AF1000, *∆yciA*::FRT	This study
DH5	F^−^ φ80*lac*ZΔM15 Δ (*lac*ZYA-*arg*F) U169 *rec*A1 *end*A1 *hsd*R17(r_K_^−^, m_K_^+^) *pho*A *sup*E44 λ^−^*thi*-1 *gyr*A96 *rel*A1	Invitrogen
pJBGT3Rx	*t3* and *rx* from *H. boliviensis* under p_lacUV5_ and lacI control (p15A/Cm)	Jarmander et al. (2015)
pBAD*zwf*	*zwf* from *E. coli* under control of p_araBAD_ (pBR22/Amp)	Perez-Zabaleta et al. (2016)
pJBG-Blank	p_lacUV5_ and *lacI* control (p15A/Cm)	This study
pBAD-(Km)-Blank	p_araBAD_ (pBR22/Km)	This study
pBAD-(Km)-*yciA*	*yciA* from *E. coli* AF1000 under control of p_araBAD_ (pBR22/Km)	This study
pBAD-(Km)-*yciA-zwf*	*yciA* and *zwf* from *E. coli* AF1000 under control of p_araBAD_ (pBR22/Km)	This study
pBAD-(Km)-*zwf-yciA*	*yciA* and *zwf* from *E. coli* AF1000 under control of p_araBAD_ (pBR22/Km)	This study
pBAD-(Km)-*zwf*	*zwf* from *E. coli* AF1000 under control of p_araBAD_ (pBR22/Km)	This study
pKD3	Template plasmid used for the FRT-flanked cat cassette.	Addgene # 45604, Datsenko and Wanner (2000)
pCmFRT*	pKD3 plasmid used with removed RBSs.	This study
pKD4	Template plasmid used for the Km cassette	Addgene #45605, Datsenko and Wanner (2000)
pSIJ8	λ red recombinase genes and flippase recombinase expression	Addgene # 68122, Jensen et al. (2015)

*E. coli* strain DH5α was used for replication of all plasmids. Plasmid construction was done by Gibson Assembly (Gibson et al. 2010, 2009) of PCR fragments obtained using Phusion DNA polymerase (Thermo Fisher Scientific, Waltham, MA) and designed primers. All plasmids were constructed using fragments and primers as indicated in Table S2 in Supplementary Material. These fragments were amplified from either strain AF1000 or respective template plasmids. Constructs were confirmed by DNA sequencing, and the resulting plasmids are listed in Table 1.

For 3HB production, the plasmid pJBGT3RX (Jarmander et al. 2015) harboring two genes from *H. boliviensis*; *t3* (acetoacetyl-CoA thiolase, WP_007111820); and *rx* (acetoacetyl-CoA reductase, WP_007111780) was used. This plasmid was constructed from pKM1D, a pACYC184-derived low-copy number plasmid with *ori* p15A, a lacUV5 promoter, the *lacI* repressor, and a chloramphenicol resistance gene. For this study, the control plasmid pJBG-Blank was constructed by assembling PCR fragments of pJBGT3RX’s backbone. Plasmids overexpressing genes *yciA* and *zwf* were based on and constructed from pBAD*zwf* (Perez-Zabaleta et al. 2016). The plasmid pBAD*zwf* is a pBAD/HisC (Invitrogen)-derived plasmid. It has the pBR22 ori, the araBAD promoter, and an ampicillin resistance gene. In view of observed ampicillin degradation in the medium, the antibiotic marker of the pBAD-based plasmids was switched from ampicillin to kanamycin. The backbone of pBAD was amplified from pBAD*zwf* (without the *zwf* gene nor the ampicillin resistance gene), genes *yciA* and *zwf* were amplified from *E. coli* AF1000, and the Km resistance gene was amplified from pKD4 (Datsenko and Wanner 2000). The control plasmid pBAD-(Km)-Blank was constructed by assembling the kanamycin resistance gene with two fragments of the back bone of pBAD*zwf*. All plasmids constructed in this study were constructed as indicated in Table S2 in Supplementary Material. All used and constructed plasmids in this study are listed in Table 1.

### Cultivation medium

The cultivation medium used was based on a heat-sterilized (121 °C for 20 min) nitrogen-restricted minimal salt medium consisting of 2 g L^−1^ (NH_4_)_2_SO_4_ (Merck, Darmstadt, Germany), 1.6 g L^−1^ KH_2_PO_4_ (VWR International, Leuven, Belgium), 0.7 g L^−1^ Na_3_C_6_H_5_O_7_·2H_2_O (Merck), 6.6 g L^−1^ Na_2_HPO_4_·2H_2_O (VWR International), and 50 μL L^−1^ antifoam B125 (BASF, Stockholm, Sweden). Heat-sterilized 15 g L^−1^ glucose (Thermo Fisher Scientific) was added separately after heat sterilization of the minimal medium. Filtered sterile (0.2 μm, VWR collection) 50 mg L^−1^ kanamycin (AppliChem Panreac, Darmstadt, Germany), 25 mg L^−1^ chloramphenicol (Sigma-Aldrich), 1 mL L^−1^ 1 M MgSO_4_·7H_2_O (Merck), and 1 mL L^−1^ trace element stock solutions were also added separately to the heat-sterilized media. The trace element stock solution consisted of 0.5 g L^−1^ CaCl_2_·2H_2_O (Merck), 16.7 g L^−1^ FeCl_3_·6H_2_O (Merck), 0.18 g L^−1^ ZnSO_4_·7H_2_O (Merck), 0.16 g L^−1^ CuSO_4_·5H_2_O (Merck), 0.15 g L^−1^ MnSO_4_·4H_2_O (Merck), 0.18 g L^−1^ CoCl_2_·6H_2_O (Merck), and 20.1 g L^−1^ Na_2_-EDTA (Merck). In the nitrogen-restricted fed-batch cultivations, 3.25 g L^−1^ (NH_4_)_2_SO_4_ and 20 g L^−1^ glucose were initially used instead. The feed solution consisted of 380 g kg^−1^ glucose, 95 g kg^−1^ (NH_4_)_2_SO_4_, 40 mL kg^−1^ of 1 M MgSO_4_·7H_2_O, and 40 mL kg^−1^ of trace element; the feed components were mixed together after separate sterilization. In the nitrogen-depleted fed-batch cultivation 7 g L^−1^ (NH_4_)_2_SO_4_ and 20 g L^−1^ glucose were initially used instead. A feed solution consisting of 500 g kg^−1^ glucose was used. In all fed-batch cultivations, 1 mL L^−1^ of sterile 1 M MgSO4 and 1 mL L^−1^ sterile trace element stock solution was added for every increase of 10 in OD_600_, before the feed phase started. Ten to 12 mL of a 500 g L^−1^ glucose solution was added when necessary to assure glucose was maintained in excess during the whole cultivations. 5 M NaOH (Merck) was used for pH titration for all cultivations.

### Cultivation procedure

All experiments were performed either in duplicate or triplicate. All recombinant *E. coli* variants were inoculated from a glycerol stock stored at − 80 °C to parallel sterile 1-L shake flask containing 100 mL of cultivation medium. The cells were cultivated overnight at 37 °C in an orbital shaker (Infors, Basel, Switzerland) at 180 rpm shaking. Subsequently, to start the experiments with an optical density at 600 nm (OD_600_) of 0.2, a calculated volume of each inoculum was harvested at 4030*g* in a floor centrifuge (Avanti J-20 XP JA12, Beckman Coulter, Palo Alto, CA) for 10 min.

Next, the cells were re-suspended in 25 mL of cultivation medium for batch experiments or in 10 mL of sterile saline to avoid cell lysis composed of 0.9% *w*/*v* NaCl (Scharlau, Barcelona, Spain) for fed-batch experiments. Afterwards, cells were used to inoculate parallel sterile 1-L stirred tank bioreactors (STR) (Greta, Belach Bioteknik, Stogås, Sweden) containing 800 mL (batch experiments) or 650 mL (fed-batch experiments). Except for the nitrogen-depleted fed-batch cultivations, cultivation medium in the STR contained 200 μM isopropyl β-D-1-thiogalactopyranoside (IPTG) (VWR International) and 0.002%(*w*/*w*) L-arabinose (Sigma-Aldrich) to induce recombinant expression. Nitrogen-depleted fed-batch cultivations were induced with 200 μM IPTG (VWR International) and 0.002%(*w*/*w*) L-arabinose (Sigma-Aldrich) when OD_600_ reached 9. The temperature was maintained at 37 °C. By adjusting the airflow and stirring speed when needed, the dissolved oxygen tension (DOT) was kept above 20% saturation for all bioreactor cultivations. The pH was maintained at 7.0 by titration with 5 M NaOH for all cultivations. Antifoam was added when required. Samples for determination of OD_600_, glucose, 3HB, acetic acid (HAc), and ammonium were withdrawn regularly during cultivations. In all cultivations, an approximate sample volume of 2.5 to 3 mL was taken out at each sampling point. Batch experiments were performed for a total of 9.5 h, nitrogen-reduced fed-batch experiments were performed for a total of 19.5 h, and nitrogen-depleted fed-batch experiments were performed for a total of 24 h.

Nitrogen-reduced fed-batch experiments were performed in 1 L bioreactors with an initial volume of 650 mL. The constant feed with reduced nitrogen was started after depletion of ammonia in the batch phase, as observed by an increase in the dissolved oxygen tension. The volumetric flow rate of the constant feed was calculated by using the following equation:

1 $$ F=\frac{\upmu \bullet {x}_o\bullet V}{S\bullet {Y}_{xs}} $$

where *F* (kg_feed_ h^−1^) is the constant feed rate, μ is the specific growth rate before feed start, *x*_o_ (g L^−1^) is the CDW at feed start, *V* (L) is the volume of medium in the reactor, *S* (g kg^−1^) is the concentration of ammonium in the feed, and *Y*_xs_*(g*_x_*g*_s_^−1^) is the yield of cells over ammonium. During the feed phase, glucose was monitored each second hour by test strips (Siemens, Bayer Uristix, Ref 2857), and when the concentration was below 5 g L^−1^, 10 mL of 500 g L^−1^ glucose was manually added to the reactor. In the ammonium-restricted fed-batch for strain AF1000 harboring plasmids, pJBGT3RX and pBAD-(Km)-*zwf* glucose was added at 7.7 h, 10.7 h and 14.5 h after inoculation and for strain AF1000 pJBGT3RX pBAD-(Km)-*zwf-yciA* glucose was added at 9.6 h and 12.7 h. A final volume of 900 mL was attained in the nitrogen reduced fed-bath experiments.

Nitrogen depleted fed-batch experiments were performed in 1 L bioreactors with an initial volume of 650 mL. The constant feed consisting of a solution of 500 g L^−1^ glucose started after depletion of the ammonia in the batch phase, as observed by an increase in the dissolved oxygen tension. The volumetric flow rate of the constant feed *F* was of 4*10^−3^ kg h^−1^. During the cultivation, 12 mL of 500 g L^−1^ glucose was manually added to the reactor, when the concentration was expected to be below 5 g L^−1^. For strain AF1000 harboring plasmids, pJBGT3RX and pBAD-(Km)-*zwf* glucose was added at 5.2 h, 7.2 h, 8.3 h, and 9.2 h after inoculation, and for strain AF1000, pJBGT3RX pBAD-(Km)-*zwf-yciA* glucose was added at 5.3 h, 7.4 h, 9.4 h, and 11.6 h after inoculation. A final volume of 745 mL was attained in the nitrogen depleted fed-bath experiments.

### Cultivation sample analysis

Cell growth was monitored by measuring the OD_600_ of cell suspensions in a spectrophotometer (Genesys 20, Thermo Scientific) after dilutions to OD_600_ between 0.1 and 0.2 in saline solution. The OD_600_ was converted to a gram per liter basis (CDW) by multiplying it by a pre-determined factor of 2.7. For measuring metabolites, cell suspension samples were centrifuged at 1700*g* in a tabletop centrifuge (Micro Star 12, VWR International) for 5 min, followed by filtering the supernatant through a syringe filter (0.2 μm, VWR International). Subsequently, the filtered supernatant samples were stored at − 20 °C until analysis. Quantification of glucose, 3HB, and acetic acid was done using ion exchange high-performance liquid chromatography (HPLC) (Alliance Waters 2695, Stockholm, Sweden) equipped with an HPX-87H organic acid column (Bio-Rad, Hercules, CA), using either a refractive index (RI) detector (Waters, 2414) at 410 nm for glucose or a UV detector (Waters, 2996) at 210 nm for organic acids with operating conditions to generate peak separation (0.5 mL min^−1^ flow rate, 0.008 N H_2_SO_4_ mobile phase, column temperature 20 °C). Ammonium concentrations were determined using the commercially available enzymatic kits: Ammonia Kit Cat No. K-AMIAR (Megazyme, Leinster, Ireland).

### Calculation of rates

For batch ammonium-depletion experiments, calculation of rates and yields was distinguished between the exponential growth phase and the ammonium-depleted phase. In the exponential growth phase, the following calculations were performed: The specific growth rate (*μ*) was obtained from the least square exponential fit of the CDW data. The yield of product with respect to CDW (*Y*_3HB/X_) was calculated as the slope obtained from plotting the variation in product concentration (*P*-*P*_o_) against the variation of CDW (*X*-*X*_o_). The specific production rate (*q*_3HB_) was calculated as the product of the yield (*Y*_3HB/X_) and the growth rate (*μ*). The same was done for calculation of the glucose-specific consumption rate (*q*_glc_), which was then used for calculation of the yield of product with respect to glucose (*Y*_3HB/Glc_) that was calculated as the quotient of the specific production rate (*q*_p_) and the specific substrate consumption rate (*q*_s_). In the ammonium-depleted phase, the CDW was considered to be constant and was calculated as the average of its values. The specific production rate (*q*_p_) was determined by fitting a linear curve (by least square regression) of 3HB concentration in function with time and dividing its first-order derivative by the CDW. The same was done for calculation of the glucose-specific consumption rate (*q*_glc_). The yield (*Y*_3HB/Glc_) was calculated as the quotient of the specific production rate (*q*_p_) and the specific substrate consumption rate (*q*_s_).

For fed-batch experiments, the productivities were calculated for the feed part of the experiment only. All calculations took into account the volume change during fed-batch fermentations. The values for total amount of cell mass (CM; g) were fitted as a function of time, Eq. (2), in the appropriate interval by a least square regression. The total amount of product, 3HB in grams, was fitted as a function of time, Eq. (3), in a similar way. The concentration of cell mass in the broth (CDW; g L^−1^), was also fitted with a function dependent on time Eq. (4), in a similar manner.

2 $$ \mathrm{CM}(t) $$

3 $$ 3\mathrm{HB}(t) $$

4 $$ \mathrm{CDW}(t) $$

The total 3HB production rate (*R*_p_; *g*_p_ h^−1^), is defined as the derivative of Eq. (3).

5 $$ {R}_{\mathrm{p}}(t)=3{\mathrm{HB}}^{\prime }(t) $$

The biomass-specific 3HB production rate (*g*_p_*g*_x_^−1^ h^−1^), Eq. (6), was calculated by dividing the total rate by the function for cell mass Eq. (2).

6 $$ {q}_{\mathrm{p}}(t)=\frac{\ {R}_{\mathrm{p}}(t)}{\mathrm{CM}(t)} $$

The volumetric rates, Eq. (7), is defined as the specific rate multiplied by the cell mas concentration in the broth in the reactor

7 $$ {r}_{\mathrm{p}}(t)={q}_{\mathrm{p}}\bullet \mathrm{CDW}(t) $$

## Results

### Deletion of thioesterase *yciA* significantly decreases 3HB production in batch experiments

**Fig. 2:**
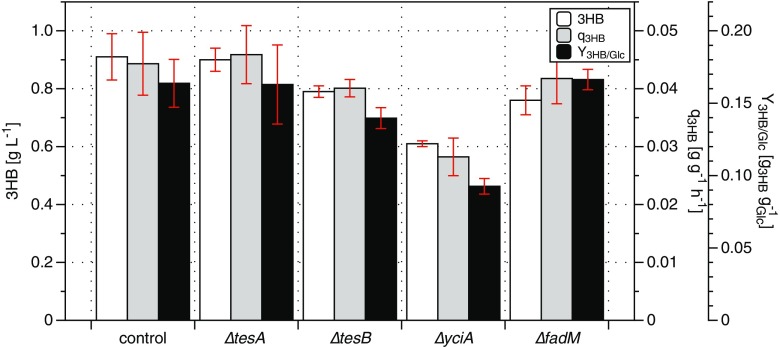
Assessment of the quantitative impact of deletion of four thioesterase-encoding genes on 3HB production by *E. coli* AF1000 expressing *t3* and *rx*. Final 3HB concentration was measured at the end of the nitrogen-depleted batch cultivation, whereas the reported specific 3HB productivity (q_3HB_) and yield of 3HB on glucose (Y_3HB/Glc_) were calculated over the nitrogen-depleted phase. Mean deviations for all strains were calculated from duplicate experiments, except for the control and Δ*tesA* strains, which were done in triplicate. The control strain was *E. coli* AF1000 expressing *t3* and *rx* without thioesterase deletions

To investigate their contribution to hydrolysis of 3HB-CoA, four genes encoding *E. coli* thioesterases *tesA, tesB, yciA*, and *fadM*, were individually deleted in the AF1000 strain background expressing the *H.boliviensis* thiolase *t3* and reductase *rx* catalyzing the first two steps of the 3HB pathway. Previous work has shown that determining 3HB production during glucose-grown batch cultivation and the following nitrogen-depleted phase, while maintaining high glucose concentrations, was a good way to screen for the impact of mutations on 3HB production (Guevara-Martínez et al. 2015; Perez-Zabaleta et al. 2016). All four deletion strains displayed a maximum specific growth rate of approximately 0.67 h^−1^, similar to the control strain. Under these conditions, the control strain produced a final 3HB titer of 0.91 g L^−1^ and showed a 3HB yield on glucose of 0.16 g g^−1^ at a specific production rate of 0.044 g g^−1^ h^−1^ during the nitrogen-depleted phase (Fig. 2). Deletion of *tesA* did not result in any significant change in either of the 3HB production parameters (Fig. 2), while deletion of *tesB* and *fadM* resulted in a modest decrease of the final 3HB concentration (Fig. 2). Deletion of *yciA* showed the biggest impact on 3HB production, with a 32% decrease in final concentrations being accompanied by a 43% decrease in yield and 36% decrease in specific production rate compared to the control strain (Fig. 2). Simultaneous with the decrease in 3HB production observed upon deletion of *yciA*, the specific productivity of acetic acid increased for the *yciA* deleted strain (0.036 g g^−1^ h^−1^) compared to the control strain (0.024 g g^−1^ h^−1^), which is in line with a decreased pull on acetyl-CoA by the 3HB pathway. The 3HB yield and specific productivities showed similar trends between the four strains during the exponential growth phase (data not shown). None of the four individual deletions completely abolished 3HB-CoA hydrolysis indicating that remaining thioesterases, potentially including ones not considered in this study (e.g., *ydiI ybgC*) (Kuznetsova et al. 2005), could still catalyze the hydrolysis. Nevertheless, for the thioesterases included in this study, the data indicate that the native *E. coli* thioesterase YciA was the largest contributor to 3HB-CoA hydrolysis in the *t3*-*rx* expressing AF1000 strain background*.*

### Overexpression of *yciA* increased 3HB production

**Fig. 3:**
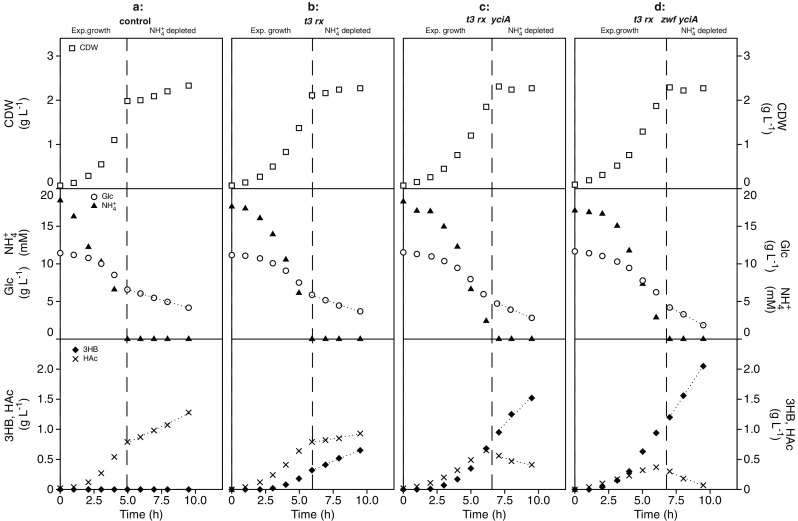
Growth, glucose consumption, and product formation during ammonium-depleted batch cultivations of *E. coli* AF1000 engineered for production of 3HB. **a** pJBG-Blank pBAD-(Km)-Blank, non-3HB-producing reference strain. **b** pJBGT3RX pBAD-(Km)-Blank, 3HB-producing control strain. **c** pJBGT3RX pBAD-(Km)-*yciA*. **d** pJBGT3RX pBAD-(Km)-*zwf-yciA*, in batch during ammonium depletion. The shown parameters are cell dry weight (CDW, open squares), glucose (Glc, open circles), ammonium (NH_4_^+^, filled triangles), acetic acid (HAc, crosses), and (*R*)-3-hydroxybutyrate (3HB, filled diamonds). 3HB, glucose, and HAc were fitted with first-order polynomials in the depleted phase. The dashed lines mark the shift between exponential growth and nitrogen depletion. This figure shows one representative replicate experiment for every strain. The remaining replicates of the set of triplicate (control, *t3-rx* and *t3-rx-yciA*) and duplicate (*t3-rx-yciA-zwf*) are included as Fig. S1 and Fig. S2 in Supplementary Material

To investigate whether overexpression of *yciA* positively influences 3HB production in engineered *E. coli*, an AF1000 strain containing pBAD-(Km)-*yciA* in addition to the production plasmid pJBGT3RX which expresses the first 2 genes encoding for the pathway towards 3HB, was screened in triplicate in nitrogen-depleted batch cultures identical to the previous screening of deletion strains (Fig. 3c). In addition, a non-3HB-producing control strain (pJBG-Blank + pBAD-(Km)-Blank; Fig. 3a) and a 3HB-producing strain without *yciA* overexpression (pJBGT3RX + pBAD-(Km)-Blank; Fig. 3b) were also tested in triplicate under the same conditions. AF1000 harboring both empty plasmids showed a growth rate of a 0.74 h^−1^ and, as expected, no production of 3HB was detected. The strain containing plasmid pJBGT3RX and the empty plasmid pBAD-(Km)-Blank showed a specific growth rate of 0.6 h^−1^. When pBAD-(Km)-*yciA* was used together with pJBGT3RX, the growth rate further decreased to 0.52 h^−1^. However, since overexpression of *yciA* alone (pBAD-(Km)-*yciA* + pJBG-Blank) resulted in a growth rate of 0.73 h^−1^ (data not shown), which is not significantly different to that of the negative control strains; this reduced growth rate is likely caused by redirection of carbon towards 3HB (see below) rather than by metabolic burden or toxicity of the overexpressed *yciA*. Independent of their maximum specific growth rates, all strains grew to approximately the same CDW (2.2 g L^−1^) at the start of the nitrogen-depleted phase.

Both strains harboring plasmid pJBGT3RX showed 3HB production in both phases (Fig. 3b and c). Overexpression of *yciA* together with genes *t3* and *rx* (Fig. 3c) doubled the final concentration of 3HB from 0.7 to 1.45 g L^−1^ compared to the strain without *yciA* overexpression (Fig. 3b). In line with the observed increase in final 3HB concentration, also the specific productivity and 3HB yield on glucose doubled upon overexpression of *yci*A in both the exponential growth and nitrogen-depleted phases (Table 2). In line with competition between 3HB and acetic acid production for the common precursor acetyl-CoA, the increase of 3HB production from negative control to *t3-rx*, and from *t3-rx* to *t3-rx-yciA*, is accompanied by a significant reduction in acetic acid formation (Fig. 3a–c). In the nitrogen-depleted phase, the *t3-rx-yciA* even consumed some of the acetic acid that was formed during the exponential phase (Fig. 3c).

**Table 2:** Calculated parameters for 3HB production in a nitrogen-depleted batch by *E. coli* and different plasmid combinations

Expressed plasmids	Phase	Growth rate	3HB titer	q_3HB_	Y_3HB/Glc_
		(h^−1^)	(g L^−1^)	(g g^−1^ h^−1^)	(g g^−1^)
pJBG-Blank pBAD-(Km)-Blank	Exp. growth	0.739 ± 0.017	n.d.	–	–
	N-depletion	–	n.d.	–	–
pJBGT3RX pBAD-(Km)-Blank	Exp. growth	0.597 ± 0.010	0.35 ± 0.03	0.102 ± 0.003	0.060 ± 0.003
	N-depletion	–	0.70 ± 0.06	0.045 ± 0.002	0.162 ± 0.010
pJBGT3RX pBAD-(Km)-*yciA*	Exp. growth	0.521 ± 0.003	0.65 ± 0.03	0.205 ± 0.011	0.120 ± 0.096
	N-depletion	–	1.45 ± 0.07	0.115 ± 0.006	0.302 ± 0.019
pJBGT3RX pBAD-(Km)-*zwf-yciA*	Exp. growth	0.477 ± 0.004	0.98 ± 0.04	0.270 ± 0.008	0.170 ± 0.012
	N-depletion	–	1.93 ± 0.12	0.153 ± 0.003	0.302 ± 0.007

*n*.*d*. no product detected

Mean deviation was calculated for triplicate experiments for all the plasmid combinations except for combination pJBGT3RX pBAD-(Km)-zwf-yciA in which the mean deviation was calculated for duplicate

Previous results have shown that increased NADPH supply by overexpression of glucose-6-phosphate dehydrogenase (*zwf*) in an *E. coli* AF1000 strain expressing *t3-rx* increased 3HB production (Perez-Zabaleta et al. 2016). To investigate whether the beneficial effect of *yciA* overexpression was additive to the effect of *zwf* overexpression, two plasmids were constructed in which *yciA* and *zwf* were expressed in the same operon: pBAD-(Km)-*zwf-yciA* and pBAD-(Km)-*yciA-zwf*. Expressing pJBGT3RX together with pBAD-(Km*)-zwf-yciA* under the same conditions further increased 3HB concentration to 1.93 g L^−1^ (Fig. 3d; Table 2). This positive effect was not observed when instead pBAD-(Km)-*yciA-zwf* was used, which gave a 3HB concentration of 1.3 g L^−1^ (data not shown), indicating that the order of these two genes in the operon had a significant effect. In line with the increased 3HB concentrations, expression of pBAD-(Km*)-zwf-yciA* also increased the specific productivity by 33% (Table 2). However, although the 3HB yield on glucose increased during the exponential growth phase, no further increase in the 3HB yield was observed during the nitrogen-depleted phase for the strain expressing pBAD-(Km*)-zwf-yciA* (Table 2).

### Fed-batch cultivations to optimize 3HB production

**Fig. 4:**
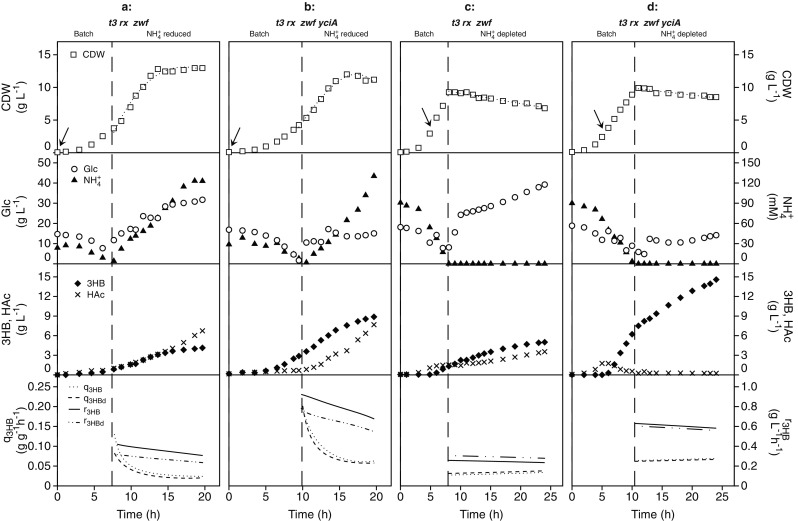
3HB production in fed-batch cultivations of *E. coli* AF1000 expressing either *t3-rx-zwf* or *t3-rx-zwf-yciA* under either ammonium-reduced or depleted conditions. **a** NH_4_^+^-reduced conditions for AF1000 harboring pJBGT3RX and pBAD-(Km)-*zwf*, reference cultivation. **b** NH_4_^+^-reduced conditions for AF1000 harboring pJBGT3RX and pBAD-(Km)-*zwf-yciA*. **c** NH_4_^+^-depleted conditions for AF1000 harboring pJBGT3RX and pBAD-(Km)-*zwf*, reference cultivation. **d** NH_4_^+^-depleted conditions for AF1000 harboring pJBGT3RX and pBAD-(Km)-*zwf-yciA*. The shown parameters are cell dry weight (CDW, open squares), glucose (Glc,open circles), ammonium (NH_4_^+^, filled triangles), acetic acid (HAc, crosses), and (*R*)-3-hydroxybutyrate (3HB, filled diamonds). One representative replicate from a set of duplicates is shown in the figure, with duplicates included as Fig. S3 in Supplementary Material. Specific production rates (q_3HB_) and volumetric rates (r_3HB_) are represented as functions obtained from least square fits of the data and are shown for both replicate experiments. All parameters were linearly fit, with the exception of CDW, which was fit with a third order polynomial. The dashed vertical line marks the shift between batch phase and fed-batch phase with feed of the respective reduced nutrient and glucose. The arrow indicates the time of induction

The observed improvements in 3HB titer, but especially in the biomass specific 3HB production rate and yield (Fig. 3; Table 2), indicate that *yciA* overexpression is likely to also have a positive effect in fed-batch bioreactor experiments designed to optimize 3HB production. Two-phase fermentations with a first batch phase to allow rapid growth of the biocatalyst, in our case up to 4–5 g/L CDW (Fig. 4), and a second fed-batch-phase with a reduced feed of one nutrient that improves product formation, are commonly used in the industry (Luzier 1992; Yamanè and Shimizu 1984). In this study, we designed constant-feed phases with reduced nitrogen in combination with glucose excess since previous work had shown this to be beneficial for 3HB production (Guevara-Martínez et al. 2015).

As a reference without *yciA* overexpression, the strain overexpressing *t3*, *rx*, and *zwf* was grown in fed-batch cultivations with reduced ammonium (Fig. 4a). At the end of the batch phase upon depletion of nitrogen, the desired CDW of 4 g L^−1^ was obtained before the feed was started. During the first 6 h of the feed phase, a linear increase of the CDW was observed as expected with a constant feed. Although glucose was maintained in excess throughout the experiment, ammonium accumulation was observed, which was caused by decreased cell growth after 6 h into the feed phase, which was likely caused by acetic acid accumulation (Fig. 4a) and/or the metabolic burden of the induced pathway or pathway proteins (Jones et al. 2000). Starting at 0.11 g g^−1^ h^−1^, the 3HB specific productivity slightly decreased throughout the feed phase, resulting in a time-averaged productivity of 0.036 g g^−1^ h^−1^ for the control strain (Fig. 4a) and an overall 3HB yield on glucose of 0.06 g g^−1^. Whereas 3HB and acetic acid were produced at similar rates during the first 8 h of feed, acetic acid was the dominant product during the last 5 h. The final titers of 3HB and acetic acid were 4.1 g L^−1^ and 6.7 g L^−1^, respectively, for the strain without *yciA* overexpression (Fig. 4a). Grown in the identical experimental set-up, the strain overexpressing *yciA* in addition to *t3*, *rx*, and *zwf* reached a CDW of 4.8 g L^−1^ at the end of the batch phase (Fig. 4b). During the feed phase, growth, nitrogen accumulation, and acetic acid production were similar as observed for the control strain. In contrast, the *yciA* overexpressing strain showed a 2.3-fold increase in the time-averaged specific 3HB productivity in the feed phase from 0.036 to 0.085 g g^−1^ h^−1^ and an accompanying increase of the overall 3HB yield from 0.06 to 0.14 g g^−1^. The final titer of 3HB doubled compared to the control strain without *yciA* overexpression to 8.9 g L^−1^, while a similar acetic acid titer of 7.7 g L^−1^ was obtained (Fig. 4b).

A nitrogen-depleted fed-batch experiment with delayed induction of the 3HB pathway (at OD_600_ 9 instead of 0.2) was designed to simultaneously avoid the metabolic pathway burden during the growth phase and enable acetic acid re-consumption, as observed during nitrogen-depleted batch experiments (Fig. 3c). Upon depletion of nitrogen at the end of the batch phase, both *t3*, *rx*, and *zwf* with and without *yciA* showed a CDW of approximately 9.5 g L^−1^ was obtained before the feed phase started (Fig. 4c and d). During the first 3 h of the feed phase, the CDW remained constant. After this, the CDW decreased linearly to a final value of 6.8 g L ^−1^ for the strain expressing *t3*, *rx*, *zwf* and 8.5 g L^−1^ for the strain expressing *t3*,*rx*, *zwf*, *yciA*. The reference strain without *yciA* overexpression (Fig. 4c) showed a time-averaged 3HB productivity of 0.035 g g^−1^ h^−1^, a 3HB yield on glucose of 0.13 g g^−1^ in the nitrogen-depleted phase, and an overall yield of 0.11 g g^−1^. The final titers of 3HB and acetic acid were 5.4 g L^−1^ and 3.5 g L^−1^ respectively. In line with the results from the nitrogen-depleted batch experiments, after induction of the 3HB pathway, the *yciA* overexpressing strain was able to re-consume acetic acid even before onset of nitrogen depletion. The HAc concentration stayed constant at 0.2 g L^−1^ during the feed phase (Fig. 4d). The time-averaged specific 3HB productivity increased from 0.036 to 0.066 g g^−1^ h^−1^ as a result of *yciA* overexpression and resulted in an increase of the 3HB yield from 0.13 to 0.24 g g^−1^ (Fig. 4c and d). The *yciA* overexpressing strain showed an overall yield of 0.21 g g^−1^ in nitrogen-depleted fed-batch cultures with a final 3HB concentration of 14.3 g L^−1^ (Fig. 4d).

## Discussion

Deletion and overexpression studies identified a clear role for thioesterase YciA in engineered 3HB-producing *E. coli* AF1000 strains. Thioesterase YciA was, however, not the sole contributing thioesterase, as illustrated by the residual 3HB production (Fig. 2). Minor contributions to 3HB productions were observed for TesB and FadM. Deletion of *tesA* had no effect on 3HB production, which is probably due to the enzyme’s periplasmic localization (Cho and Cronan 1993). To assess the involvement of other native thioesterases not considered for this study in future research, a control strain containing the deletions in *tesA*, *tesB*, *fadM*, and *yciA* would be beneficial.

The selected YciA has shown to have catalytic efficiency towards many intermediates including acetyl-CoA, acetoacetyl-CoA, both configurations of 3HB, crotonate, and butyrate (Clomburg et al. 2012). Despite previous in vitro measurements showing that YciA exhibits twice as much catalytic efficiency towards acetyl-CoA than towards 3HB-CoA (Clomburg et al. 2012), overexpression of *yciA* in this study did not result in increased acetic acid formation while doubling 3HB production. This likely reflects the efficient pull by the combined 3HB pathway on the acetyl-CoA pool, and overexpression of *yciA* directed even more carbon flux towards product formation by reducing the concentration of the 3HB-CoA intermediate. This is also illustrated by the decreased formation, and even re-consumption, of acetic acid during nitrogen-depleted batch cultures (Fig. 2c–d). The importance of the pathway preceding the thioesterase is illustrated by the observations by (Clomburg et al. 2012), who by knocking out *yciA* in *E. coli* strain JC01 (MG1655), while overexpressing reverse β-oxidation enzymes responsible for (S)-3HB-CoA formation, demonstrated a large contribution of YciA in (S)-3HB-CoA hydrolysis, but in their context overexpression of *yciA* actually eliminated 3HB production and instead increased acetic acid formation. A main difference is that in this present study, YciA was preceded by *H. boliviensis*’ thiolase and reductase instead of the reverse β-oxidation, which might explain the contradicting findings of increased 3HB production in *E. coli* AF1000. This clearly illustrates the importance of improved understanding of the spectrum of thioesterases in different *E. coli* strains, beyond the commonly used TesB (Liu et al. 2007). Although directly relevant for the thioesterase selection and specificity for 3HB production, metabolic engineering efforts for the production of other compounds, that either require thioesterase activity or have acyl-CoA intermediates (Handke et al. 2011; McMahon and Prather 2014), can benefit from strain-specific information to minimize formation of by-products.

Overexpression of *yciA* for hydrolyzing (*R*)-3-hydroxybutyryl-CoA to form 3HB and design of a nitrogen-depleted fed-batch prevented acetic acid accumulation and enabled a final 3HB titer of 14.3 g L^−1^ within 24 h in a one-stage fermentation by *E. coli*. The highest observed 3HB yield on glucose was 0.3 g g^−1^, which corresponds to 40% of the maximum theoretical yield (defined as all available electrons ending up in the product and no growth) or 52% of the biochemical maximum yield with two acetyl-CoA derived from pyruvate being converted to 3HB (Table 2). This yield was achieved during the nitrogen-depleted phase of the batch cultures. However, the biomass-specific 3HB production rate under these conditions was 0.15 g g^−1^ h^−1^, which was only roughly half the rate of 0.27 g g^−1^ h^−1^ observed during exponential growth of the *yciA* and *zwf* overexpressing strain. Based on these observations, two clear targets for further strain and/or process improvement can be identified. First, formation of acetic acid as a by-product both pulls carbon and electrons away from the desired product, as well as precludes the efficient continuation of the cultivation at concentrations above ± 5 g L^−1^ due to its inhibitory effect on cell growth and metabolism. Despite successfully avoiding acetic acid accumulation in nitrogen-depleted fed batch cultures (Fig. 4d), reduction of acetic acid by either further strain engineering or through further optimization of fermentation processes therefore seems a logical target. A second point for improvement is further uncoupling between growth and product formation, or more precisely the specific growth rate and the specific product formation rate. In this study, the highest yields coincided with low rates, such as during nitrogen depletion, while the highest rates were observed during exponential growth. This uncoupling could for instance be achieved by optimized fermentation protocols with tighter control of the limiting nutrients and also by further deregulating the coupling between growth and the supply of acetyl-CoA and NADPH.

## Electronic supplementary material

ESM 1 (PDF 285 kb)
