# Supplementary material for: The role of the acyl-CoA thioesterase “YciA” in the production of (R)-3-hydroxybutyrate by recombinant Escherichia coli
Source: Appl Microbiol Biotechnol. 2019 Mar 5;103(9):3693–704. doi: 10.1007/s00253-019-09707-0 (PMC6469607; doi:10.1007/s00253-019-09707-0)
Supplement: Supplementary file 1 — (PDF 285 kb) [file 253_2019_9707_MOESM1_ESM.pdf]

Supplementary material

The role of *yciA* in the production of (*R*)-3-hydroxybutyrate by recombinant *Escherichia coli*

Applied Microbiology and Biotechnology

Mónica Guevara-Martínez [1, 2], Mariel Perez-Zabaleta[1, 2], Martin Gustavsson[1], Jorge Quillaguamán[2], Gen Larsson[1], and Antonius J.A. van Maris[1]

1 Department of Industrial Biotechnology, School of Engineering Sciences in Chemistry, Biotechnology and Health, KTH Royal Institute of Technology, Stockholm, Sweden,

2 Faculty of Science and Technology, Center of Biotechnology, Universidad Mayor de San Simón, Cochabamba, Bolivia

***Corresponding author:** Antonius J. A. van Maris, E-mail: tonvm@kth.se**,** Telephone number: +46-(0) 8-7909992.

Table S1 Primers used in this study for gene deletion. Primers were used to amplify the FRT-flanked cat cassette with homology parts from the gene of interest

Target gene	Primer sequence
AF1000 Δ*tesA*	Forward primer:<br>5’-GTGAGGATGGAGAGTTCATGCTCCCCCTGACCGACGGACTTCTTAAGATGTTGTGTAGGCTGGAGCTGCTTC-3’<br>Reverse primer:<br>5’-TAACTTTACCTGTCAGGATCTCCGTTGCTTTATGAGTCATGATTTACTAAGCCGTTGTGAACTCAGTTAGCTAGAC-3’
AF1000 Δ*tesB*	Forward primer:<br>5’-TCAACTCACTTTGGCTTGCTGCGGCAGCTTTGTTACTGGAGAGTTATATGTTGTGTAGGCTGGAGCTGCTTC-3’<br>Reverse primer:<br>5’-CCGGACGGTTTTCACCTCCGGCTATTTTTTTAATTGTGATTACGCATCACGCCGTTGTGAACTCAGTTAGCTAGAC -3’
AF1000 *ΔfadM*	Forward primer:<br>5’-TACCAGTTATGACCTCTGTACTTATAACAACAACGTAAGGTTATTGCGCTTTGTGTAGGCTGGAGCTGCTTC-3’<br>Reverse primer:<br>5’-ATGCGGCTTGAGCATCCGGCACCACAAAACGTTTACTTAACCATCTGCTCGCCGTTGTGAACTCAGTTAGCTAGAC-3’
AF1000 *ΔyciA*	Forward primer:<br>5’-AAATCATAGTAGCATCGCGCCTGTGATTTTCCTTTTAAGTCGGTTTTACCTTGTGTAGGCTGGAGCTGCTTC-3’<br>Reverse primer:<br>5’-CTCCGACCGGAGGCTTTTGACTATTACTCAACAGGTAAGGCGCGAGGTTTGCCGTTGTGAACTCAGTTAGCTAGA-3’

Table S2 Primers used in this study for plasmid construction. Primers were used to amplify genes of interest and vector backbones

Plasmid construction	Fragment name/template	Primer sequence
pJBG	F1JBG/pJBG*T3RX*	5’-GATTTCCGGCAGTTTCTACACATAT-3’<br>5’-ATTAATCGGCCGTTATTATTTGTAGGATTTACCTCCTAGCTTTCCTGTGTG-3’
	F2JBG/pJBG*T3RX*	5’-ATATGTGTAGAAACTGCCGGAAATC-3’<br>5’-ACACAGGAAAGCTAGGAGGTAAATCCTACAAATAATAACGGCCGATTAATTCACC-3’
pBAD(km)-Blank	F1BADkm/pKD4	5’-GATGAATGTCAGCTACTGGGC-3’<br>5’-AATCAATCTAAAGTATATATGAGTAAACTTGGTCTGACAGTCAGAAGAACTCGTCAAGAAGG-3’
	F2BADkm/pBAD*zwf*	5’-CTGTCAGACCAAGTTTACTCATA-3’<br>5’-CCAAAACAGCCAAGCTGGAGACCGTTTAAACTCATTAATGATGATGATGATGATGCATGTTAATTCCTCCTGTTAGCCCA-3’
	F3BADkm/pBAD*zwf*	5’-TAA TGAGTTTAAACGGTCTCCAGC-3’<br>5’-CCAGATAGCCCAGTAGCTGACATTCATC CAAAAGAGTTTGTAGAAACGCA-3’
pBAD(km)-*yciA*	F1BADkm-yciA/pKD4	5’-GATGAATGTCAGCTACTGGGC-3’<br>5’-AATCAATCTAAAGTATATATGAGTAAACTTGGTCTGACAGTCAGAAGAACTCGTCAAGAAGG-3’
	F2BADkm-yciA /pBAD*zwf*	5’-CTGTCAGACCAAGTTTACTCATA-3’<br>5’-CCAAAACAGCCAAGCTGGAGACCGTTTAAACTCATTAATGATGATGATGATGATGCATGTTAATTCCTCCTGTTAGCCCA-3’
	F3BADkm-yciA /pBAD*zwf*	5’-TAATGAGTTTAAACGGTCTCCAGC-3’<br>5’-CCAGATAGCCCAGTAGCTGACATTCATC CAAAAGAGTTTGTAGAAACGCA-3’
	F4BADkm-yciA /*E. coli* AF1000	5’-TCTACAACACATAACGTCCCTC-3’<br>5’-CATCCGCCAAAACAGCCAAGCTGGAGACCGTTTAAACTCA TTACTCAACAGGTAAGGCGC-3’
pBAD(km)-*yciA-zwf*	F1BADkm-*yciA-zwf* /pBAD(km)	5’-TAATGAGTTTAAACGGTCTCCAGC -3’<br>5’-ACAAGATCGCCCTGAGGGACGTTATGTGTTGTAGACATGTTAATTCCTCCTGTTAGCCCA-3’
	F2BADkm-*yciA-zwf* / *E. coli* AF1000	5’-ATGCATCATCATCATCATCATGCGGTAACGCAAACAGC-3’<br>5’-CGCCAAAACAGCCAAGCTGGAGACCGTTTAAACTCATTACTCAAACTCATTCCAGGAACG -3’
	F3BADkm-*yciA-zwf* / *E. coli* AF1000	5’-TCTACAACACATAACGTCCCTC-3’<br>5’- CCTGGGCTGTTTGCGTTACCGCATGATGATGATGATGATGCATGTTAATTCCTCTATAATTTACTCAACAGGTAAGGCGC-3’
pBAD(km)-*zwf-yciA*	F1BADkm-*zwf-yciA* /pBAD(km)-*yciA*	5’-ATGCATCATCATCATCATCATTCTACAACACATAACGTCC-3’<br>5’-TGACCAGGTCACAGGCCTGGGCTGTTTGCGTTACCGCCATGTTAATTCCTCCTGTTAGCC-3’
	F2BADkm-*zwf-yciA* / *E. coli* AF1000	5’-ATGGCGGTAACGCAAACA-3’<br>5’-GACGTTATGTGTTGTAGAATGATGATGATGATGATGCATGTTAATTCCTCTATAATTTACTCAAACTCATTCCAGGAACG -3’
pBAD(km)-*zwf*	F1BADkm-*zwf* /pBAD(km)	5’-TAATGAGTTTAAACGGTCTCCAGC -3’<br>5’-TGACCAGGTCACAGGCCTGGGCTGTTTGCGTTACCGCCATGTTAATTCCTCCTGTTAGCC-3’
	F2BADkm-*zwf* /*E. coli* AF1000	5’-ATGGCGGTAACGCAAACA -3’<br>5’- CGCCAAAACAGCCAAGCTGGAGACCGTTTAAACTCATTACTCAAACTCATTCCAGGAACG -3’

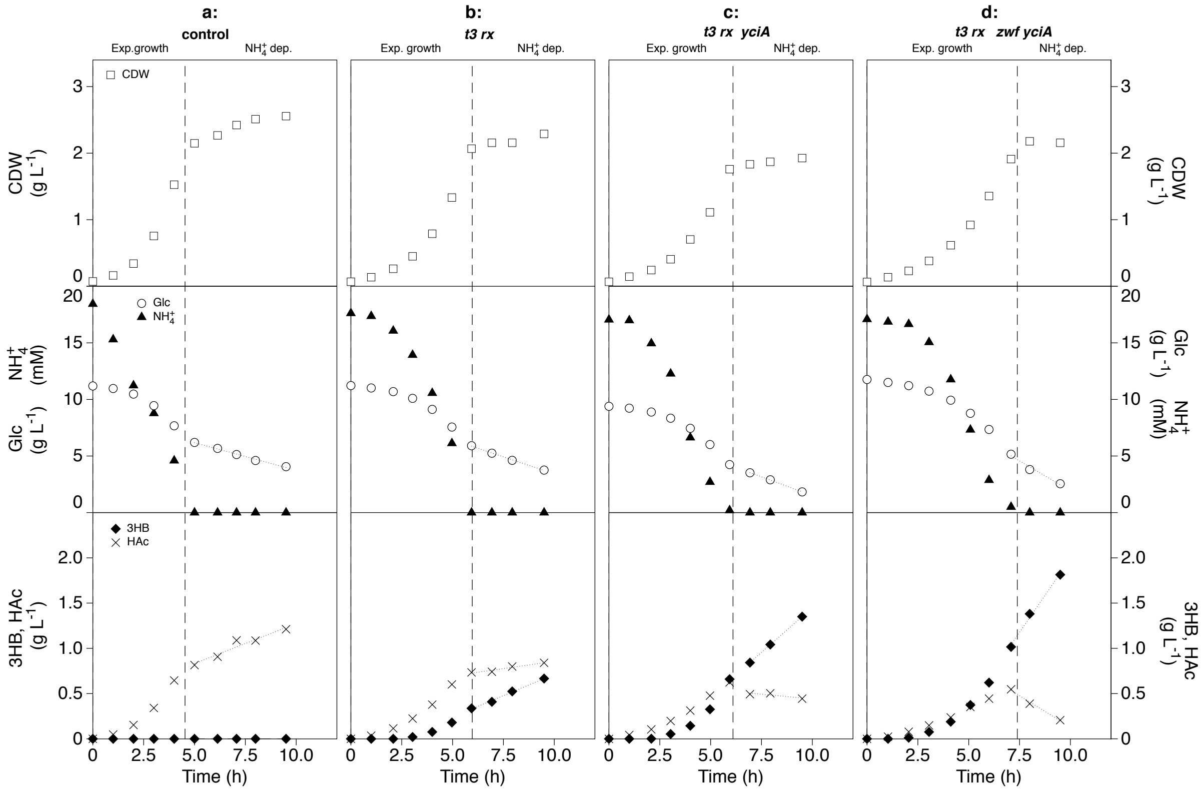

Fig. S1 Growth, glucose consumption and product formation during ammonium-depleted batch cultivations of *E. coli* AF1000 engineered for production of 3HB. **a** pJBG-Blank pBAD(km)-Blank, non-3HB producing reference strain. **b** pJBGT3RX pBAD(km)-Blank, 3HB producing control strain. **c** pJBGT3RX pBAD(km)-*yciA*. **d** pJBGT3RX pBAD(km)-*zwf-yciA*, in batch during ammonium depletion. The shown parameters are: cell dry weight (CDW, open squares), glucose (Glc, open circles), ammonium (NH_4^+, filled triangles), acetic acid (HAc, crosses) and (*R*)-3-hydroxybutyrate (3HB, filled rhombi). 3HB, glucose and HAc were fitted with first order polynomials in the depleted phase. The dashed lines mark the shift between exponential growth and nitrogen depletion. This figure shows the second replicate experiment for every strain (control, *t3-rx* and *t3-rx-yciA*, *t3-rx-yciA-zwf*)

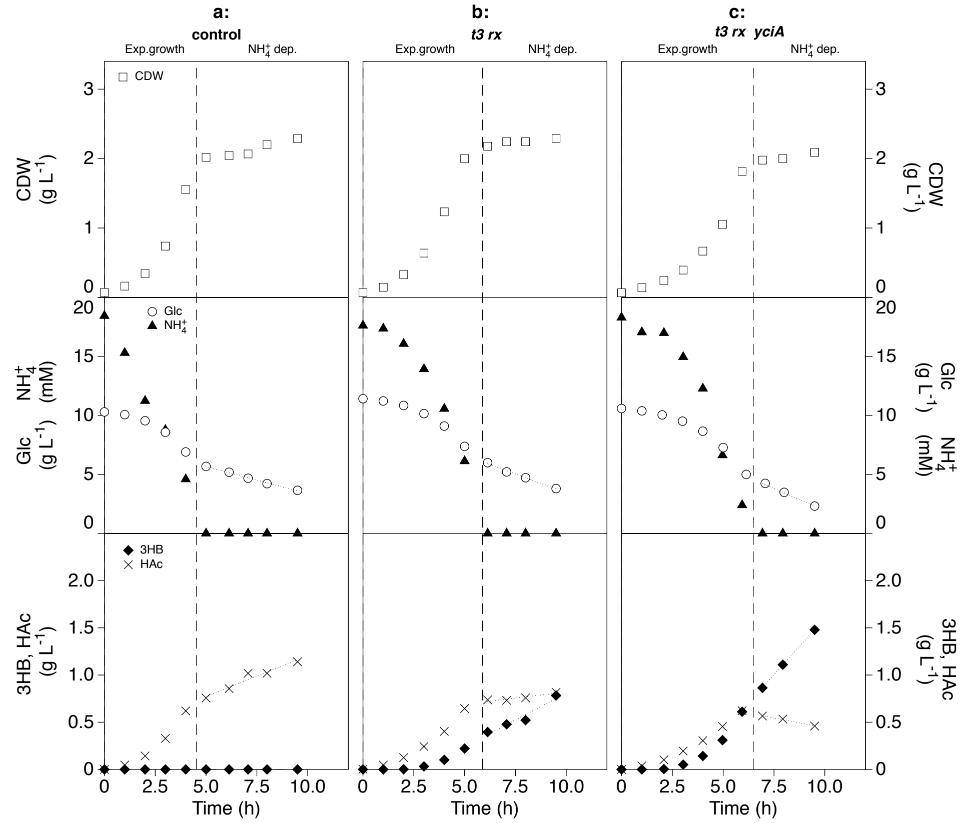

Fig. S2 Growth, glucose consumption and product formation during ammonium-depleted batch cultivations of *E. coli* AF1000 engineered for production of 3HB. **a** pJBG-Blank pBAD(km)-Blank, non-3HB producing reference strain. **b** pJBGT3RX pBAD(km)-Blank, 3HB producing control strain. **c** pJBGT3RX pBAD(km)-*yciA*, in batch during ammonium depletion. The shown parameters are: cell dry weight (CDW, open squares), glucose (Glc, open circles), ammonium (NH_4^+, filled triangles), acetic acid (HAc, crosses) and (*R*)-3-hydroxybutyrate (3HB, filled rhombi). 3HB, glucose and HAc were fitted with first order polynomials in the depleted phase. The dashed lines mark the shift between exponential growth and nitrogen depletion. This figure shows the third replicate experiment for strains: control, *t3-rx* and *t3-rx-yciA*

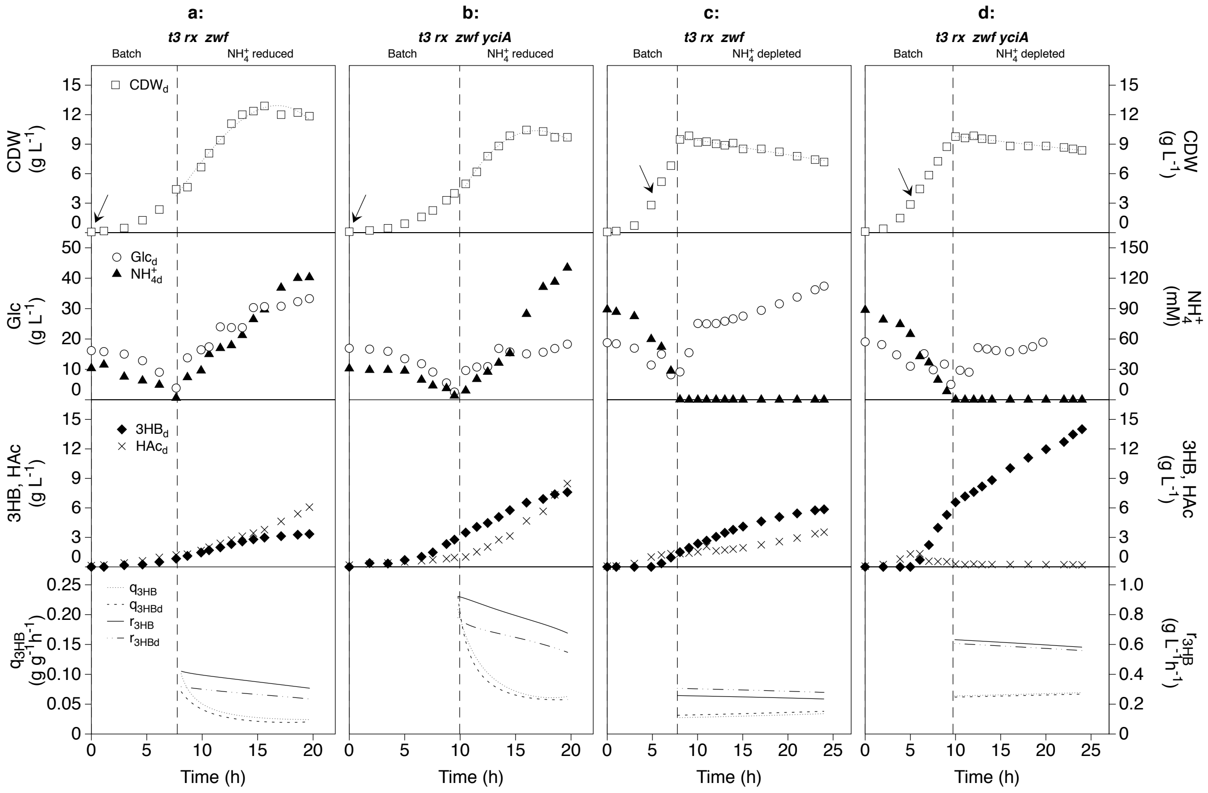

Fig. S3 3HB production in fed-batch cultivations of *E. coli* AF1000 expressing either *t3-rx-zwf* or *t3-rx-zwf-yciA* under either ammonium reduced or -depleted conditions. **a** NH_4^+-reduced conditions for AF1000 harboring pJBGT3RX and pBAD(Km)-*zwf*, reference cultivation. **b** NH_4^+-reduced conditions for AF1000 harboring pJBGT3RX and pBAD(Km)-*zwf-yciA*. **c** NH_4^+-depleted conditions for AF1000 harboring pJBGT3RX and pBAD(Km)-*zwf*, reference cultivation. **d** NH_4^+-depleted conditions for AF1000 harboring pJBGT3RX and pBAD(Km)-*zwf-yciA*. The shown parameters are: cell dry weight (CDW, open squares), glucose (Glc,open circles), ammonium (NH_4^+, filled triangles), acetic acid (HAc, crosses) and (*R*)-3-hydroxybutyrate (3HB, filled diamonds). The second representative replicate from a set of duplicates is shown in the figure. Specific production rates (q_{3HB}) and volumetric rates (r_{3HB}) are represented as functions obtained from least square fits of the data and are shown for both replicate experiments. All parameters were linearly fit, with the exception of CDW, which was fit with a third order polynomial. The dashed vertical line marks the shift between batch phase and fed-batch phase with feed of the respective reduced nutrient and glucose. The arrow indicates the time of induction
